# The impact of acupuncturists’ experience level on the efficacy of acupuncture for functional dyspepsia: an exploratory secondary analysis of a randomized clinical trial

**DOI:** 10.3389/fmed.2026.1834168

**Published:** 2026-06-11

**Authors:** Jianzhen Jiang, Chongkai Luo, Yangke Mao, Tao Yin, Zilei Tian, Jingya Cao, Xueping Yu, Tingting Ma, Hui Zheng, Xiaoping Tian, Lei Lan, Xiaohui Dong, Fanrong Liang, Fang Zeng

**Affiliations:** 1 Acupuncture and Tuina School, The 3rd Teaching Hospital, Chengdu University of Traditional Chinese Medicine, Chengdu, Sichuan, China; 2 Acupuncture and Brain Science Research Center, Chengdu University of Traditional Chinese Medicine, Chengdu, Sichuan, China; 3 Key Laboratory of Acupuncture for Senile Disease (Chengdu University of TCM), Ministry of Education, Chengdu, Sichuan, China; 4 Sichuan College of Traditional Chinese Medicine, Mianyang, Sichuan, China; 5 Hospital of Chengdu University of Traditional Chinese Medicine, Chengdu, Sichuan, China; 6 School of Nursing, Chengdu University of Traditional Chinese Medicine, Chengdu, Sichuan, China

**Keywords:** acupuncture, acupuncturist experience, functional dyspepsia, randomized clinical trial, secondary analysis

## Abstract

**Introduction:**

Acupuncture has shown significant therapeutic effects for functional dyspepsia (FD) in clinical trials. However, clinical observations reveal substantial variations in patient responses to acupuncture. In addition to patient-related factors, such variability may also be associated with differences in acupuncturists’ clinical experience. This study compared the treatment outcomes for FD between junior and senior acupuncturists.

**Materials and methods:**

This exploratory secondary analysis used data from a previous randomized controlled trial. Patients were divided into two groups based on acupuncturists’ years of practice as defined by the professional title evaluation rules, using 5 years as the cut-off (Group A treated by junior acupuncturists and Group B treated by senior acupuncturists). Outcome measures included the Symptom Index for Dyspepsia (SID), the four cardinal symptoms of FD, and the Nepean Dyspepsia Life Quality Index (NDLQI). Assessments were conducted at baseline, treatment weeks 2 and 4, and follow-up weeks 4 and 12. Data were analyzed using generalized estimating equations.

**Results:**

Adjusted analyses were performed after including items with between-group differences as covariates. The study revealed that Group B had lower SID scores than Group A at both follow-up time points—at 4 weeks (mean difference: 0.6, *p* = 0.007) and at 12 weeks (mean difference: 0.8, *p* = 0.001). In addition, Group B showed greater improvements in postprandial fullness and early satiety at both follow-ups (*p* < 0.010). However, no significant between-group differences were observed for epigastric pain or epigastric burning. Regarding quality of life, Group B also had higher NDLQI scores at the 12-week follow-up (mean difference: −2.9; *p* = 0.041).

**Conclusion:**

Based on the 5-year administrative cutoff criterion, this exploratory secondary analysis found a potential association between acupuncturists’ years of practice and acupuncture efficacy for FD, but prospective studies are needed for confirmation.

## Introduction

Acupuncture has shown significant therapeutic effects for numerous diseases and is widely recognized internationally (1, 2). However, as a complex technique performed manually by acupuncturists, its therapeutic effects are closely related to many factors (3). Previous literature has suggested that practitioner-related factors may influence acupuncture outcomes, but the evidence remains mixed, and the specific contribution of acupuncturists characteristics has not been clearly established (4–7). The level of practice experience is often considered a comprehensive reflection of various acupuncturists characteristics; however, researchers hold divergent views on how it may affect treatment outcomes (8–12). Currently, direct evidence linking acupuncturist experience level to acupuncture outcomes remains limited (13).

Functional dyspepsia (FD) is one of the most prevalent functional gastrointestinal disorders, affecting over 20% of the population. According to the Rome IV criteria, FD is diagnosed based on the presence of one or more of the following symptoms—postprandial fullness, early satiety, epigastric pain, and epigastric burning—in the absence of any structural disease detectable by imaging or endoscopy. These symptoms may be severe enough to interfere with daily activities (14, 15). In recent years, numerous studies have shown that acupuncture, an effective non-pharmacological treatment, has positive therapeutic effects on FD (16, 17). In our previous work, we conducted a multicenter, randomized clinical trial of acupuncture for FD. This trial evaluated the efficacy of different acupuncture prescriptions, sham acupuncture, and itopride in treating FD. The results demonstrated that acupuncture significantly improved symptoms and quality of life in patients with FD (17).

In clinical practice, we have observed differences in therapeutic outcomes among patients with similar symptom profiles and disease assessments when treated by acupuncturists with different levels of experience. Some patients experience marked symptom improvement with long-term efficacy, while others show suboptimal therapeutic responses or transient benefits. This observation suggests that beyond patient-specific variations, the experience level of acupuncturists may be a significant factor influencing the effectiveness of acupuncture treatment for FD. However, current research evidence on this is limited. Therefore, this study conducted an exploratory secondary analysis based on data from our previous clinical trial to examine whether acupuncturist experience level is associated with differences in symptom improvement and quality of life in patients with FD.

## Methods

### Overview of original research

#### Basic information

The original study (17) was a multicenter, randomized, controlled trial at 8 hospitals in China from April 2008 to October 2009. It aimed to investigate the efficacy and safety of acupuncture for FD patients, compared with the sham acupuncture group and the itopride group. The detailed protocol has been described in our published article. This study protocol was approved by the Ethics Committee, and the trial was conducted in compliance with the Declaration of Helsinki. Every participant was well informed and signed the informed consent form. The trial was registered at ClinicalTrials.gov (Clinical Trial Number: NCT00599677, Registration Date: 2008-01-24).

#### Participants

All participants met the diagnostic criteria for FD. Their ages ranged from 18 to 65 years. All participants had not received treatment with prokinetic drugs for 15 days before enrollment. Furthermore, they had not received other acupuncture treatments within 30 days before enrollment.

#### Grouping and intervention

A total of 720 participants were centrally randomized to one of six groups: 4 acupuncture groups, a sham acupuncture group, and an itopride group. The study included a 1-week run-in period, a 4-week treatment period, and a 12-week follow-up period. The acupuncture groups and sham acupuncture group received treatment for 5 consecutive days each week, for a total of 20 sessions. The itopride group received itopride orally according to the standard regimen. In the acupuncture groups, twirling and lifting/thrusting techniques were employed to elicit *deqi*. Following *deqi*, an electroacupuncture device was connected. Among the 480 patients assigned to the 4 acupuncture groups (Table 1, detailed information on the four acupuncture prescriptions was provided in the Supplementary material), 436 FD patients completed the 4 weeks of acupuncture treatment.

**Table 1:** Core acupoints prescriptions of the four acupuncture groups in the original RCT study.

Group	Acupoints
A	ST42, ST40, ST36, ST34 (specific acupoints on the stomach meridian)
B	ST38, ST35, ST33, ST32 (non-specific acupoints on the stomach meridian)
C	BL21, CV12 (specific acupoints commonly used for FD)
D	GB40, GB37, GB36, GB34 (acupoints on the gallbladder meridian)

#### Outcome measures

Symptom Index of Dyspepsia (SID), four cardinal symptoms of FD, and Nepean Dyspepsia Life Quality Index (NDLQI) were collected at baseline, the end of 2 weeks and 4 weeks of treatment, and at the end of 4 weeks and 12 weeks of follow-up. The SID focuses on four cardinal symptoms of FD, namely postprandial fullness, early satiety, epigastric pain, and epigastric burning. Each symptom is then graded as asymptomatic (0 point), mild (1 point), moderate (2 points), or severe (3 points). The SID score is the sum of the four cardinal symptoms scores in FD patients. The four cardinal symptom scores refer to the respective scores for postprandial fullness, early satiety, epigastric pain, and epigastric burning. The 25-item NDLQI measured the dyspepsia-specific health-related quality of life in FD patients from the following four domains: the interference (13 items), the knowledge/control (7 items), the eat/drink (3 items), and the sleep/disturb (2 items) (18).

### Secondary analysis design

#### Secondary grouping

The patients in this study were regrouped based on the treating acupuncturist rather than being originally randomized according to practitioner experience. A total of 436 FD patients from 4 acupuncture groups were included in this secondary analysis. According to the Basic Standards for Professional Title Evaluation of Healthcare Professionals issued by the National Health Commission of the People’s Republic of China (19), acupuncturists with less than 5 years of acupuncture practice were categorized as the junior experience group, whereas those with 5 years or more were categorized as the senior experience group. Subsequently, the FD patients treated by junior acupuncturists were defined as the junior acupuncturist treatment group (Group A), while those treated by senior acupuncturists were defined as the senior acupuncturist treatment group (Group B). In particular, it should be noted that the 5-year cutoff is merely an administrative grouping based on professional title evaluation rules, and does not represent a direct measure of clinical competence.

#### Observation metrics

The SID was used to assess overall dyspeptic symptoms, the four cardinal symptoms of FD to assess each symptom, and the NDLQI to evaluate quality of life in FD patients. All outcomes were assessed at baseline, week 2 of treatment, week 4 of treatment, week 4 of follow-up, and week 12 of follow-up.

#### Exploratory subgroup analysis

FD is commonly classified into postprandial distress syndrome (PDS) and epigastric pain syndrome (EPS) (20). PDS is characterized by postprandial fullness and early satiety, whereas EPS presents with epigastric pain and burning (21). However, considerable overlap between PDS and EPS is frequently observed in clinical practice, with studies reporting that 66% of patients with FD meet the diagnostic criteria for both subtypes simultaneously (22). To further explore whether acupuncturists with different experience levels exert differential effects on distinct symptom domains of FD, we conducted subgroup analysis based on PDS- and EPS-related symptom domains rather than mutually exclusive clinical subtype classifications.

After excluding 23 patients with symptom scores of 0 for both postprandial fullness and early satiety, 413 patients were included in the postprandial symptom-domain subgroup analysis (PDS-domain group: patients with at least one baseline symptom of either postprandial fullness or early satiety). Similarly, after excluding 103 patients with symptom scores of 0 for both epigastric pain and epigastric burning, 333 patients were included in the epigastric symptom-domain subgroup analysis (EPS-domain group: patients with at least one baseline symptom of either epigastric pain or epigastric burning). Because these analytic subsets were defined by the presence of specific symptom domains rather than as mutually exclusive clinical subtypes, overlap between the subsets was possible.

#### Statistical analysis

IBM SPSS 27.0 software (IBM Statistics, Armonk, NY, United States) was used for statistical analysis. GraphPad Prism 10 (GraphPad Software, Boston, MA, United States) was used for plotting.

Descriptive statistics were used to summarize demographic and clinical characteristics. Baseline univariate analyses were performed on the data for the aforementioned variables between the two groups. *Chi*-square tests were used to compare categorical variables (Fisher’s exact test was used for categorical variables with cell sizes less than 5). Independent *t*-tests or Mann–Whitney U tests were used to analyze continuous variables.

Because the grouping of FD patients in this secondary analysis study was not randomized, we included baseline differences between groups as covariates. Generalized Estimating Equations (GEE) were used to analyze repeated outcome data across time points. The models included the main effects of group and time, as well as the group-by-time interaction. Bonferroni correction was applied to the *post hoc* pairwise comparisons reported for the longitudinal outcome analyses. A two-sided *p* value <0.05 was considered statistically significant. This secondary analysis included FD patients who completed the 4-week treatment and follow-up (those who discontinued treatment were excluded), as well as those who completed treatment but dropped out during the follow-up period, for whom missing data was imputed using the last observation carried forward method.

## Results

### Characteristics of acupuncturists

A total of 12 acupuncturists participated in the acupuncture procedures, including 5 junior acupuncturists and 7 senior acupuncturists. The characteristics of the acupuncturists are summarized in Table 2. No significant differences were found in sex distribution (*p* = 0.205), number of patients treated (*p* = 0.684), or study site distribution (*p* = 0.886). Because the acupuncturists were grouped according to a 5-year cut-off, significant differences were observed inevitably between the two types of acupuncturists in age (24.8 ± 0.84 vs. 31.9 ± 5.6 years, *p* = 0.004), years of acupuncture practice (2.4 ± 0.5 vs. 9.4 ± 5.3 years, *p* = 0.004), and education level (*p* = 0.023). Specifically, all junior acupuncturists held a bachelor’s degree (100%), senior acupuncturists predominantly held advanced degrees (57.1% master’s and 28.6% doctoral degrees).

**Table 2:** Characteristics of acupuncturists.

Characteristic	Junior acupuncturist (*n* = 5)	Senior acupuncturist (*n* = 7)	*P* value
Age, years	24.8 (0.84)	31.9 (5.6)	0.004
*Sex*			0.205
Female	5 (100%)	4 (57.1%)	
Male	0 (0.0%)	3 (42.9%)	
Acupuncture practice, years	2.4 (0.5)	9.4 (5.3)	0.004
Patients treated, numbers	42.0 (58.0)	32.3 (33.6)	0.684
Education level			0.023
Bachelor’s degree	5 (100%)	1 (14.3%)	
Master’s degree	0 (0.0%)	4 (57.1%)	
Doctoral degree	0 (0.0%)	2 (28.6%)	
Study site			0.886
Site A	1 (20.0%)	2 (28.6%)	
Site B	3 (60.0%)	3 (42.9%)	
Site C	1 (20.0%)	2 (28.6%)	

Data are mean (SD) or *n* (%).

### Baseline characteristics of FD patients

**Figure 1:**
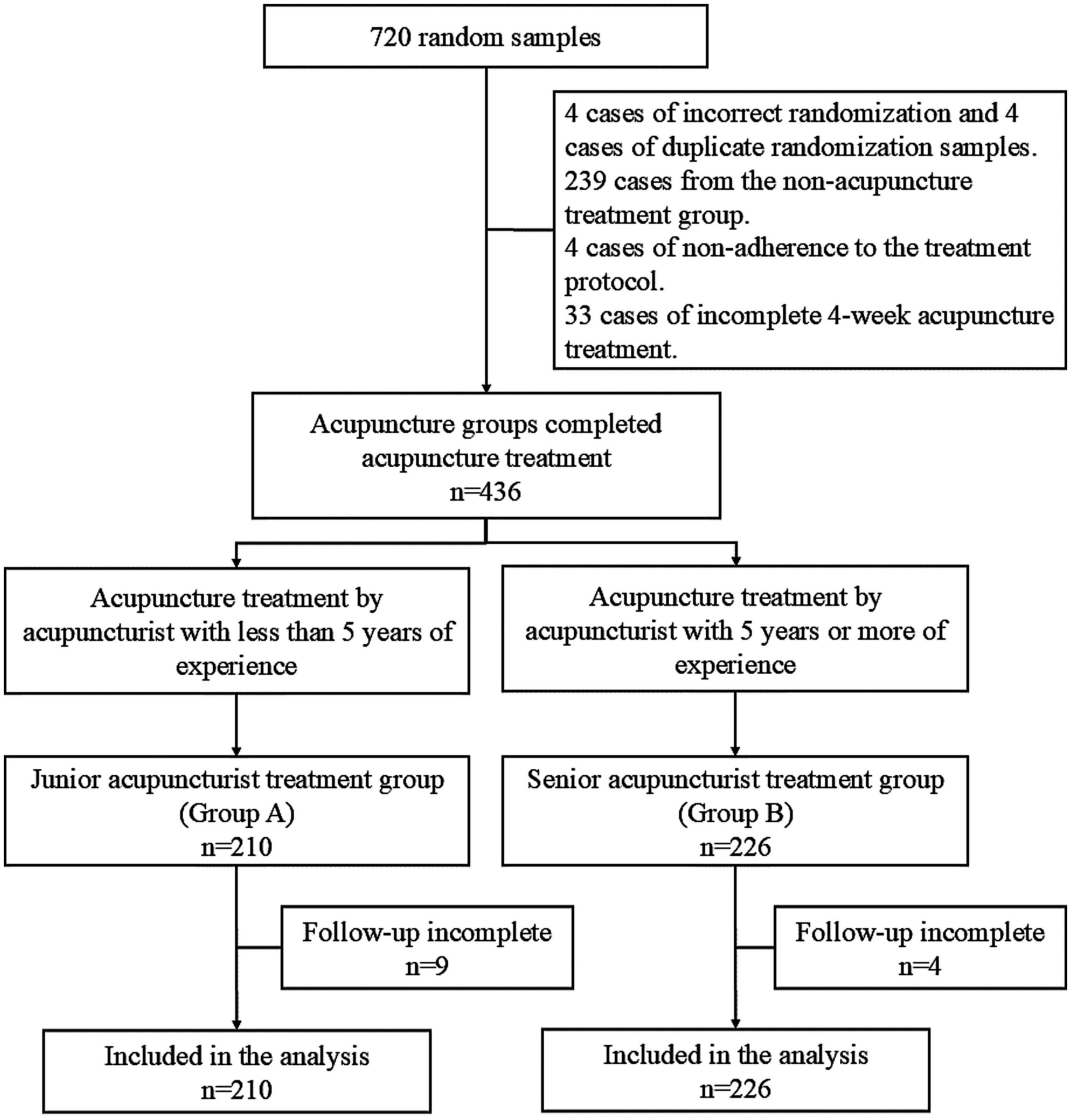
Grouping flow chart of FD patients. The samples that did not complete the follow-up were also included in the analysis by supplementing the data using the last observation carry-forward method.

Among these 436 FD patients who completed acupuncture treatment, 210 participants were treated by junior acupuncturists and were assigned to Group A, 226 participants were treated by senior acupuncturists and were assigned to Group B (Figure 1).

Baseline characteristics and baseline symptom measures of participants are presented in Table 3. No significant baseline characteristics differences were observed in age, sex distribution, body mass index (BMI), disease duration, dyspepsia subtypes (*p* > 0.05).

**Table 3:** Characteristics and symptoms measures of FD patients at baseline.

Characteristic	Group A (*n* = 210)	Group B (*n* = 226)	*P* value
Demographics			
Age, years	37.9 (13.9)	36.3 (13.2)	0.206
*Sex*			0.134
Female	155 (73.8%)	152 (67.3%)	
Male	55 (26.2%)	74 (32.7%)	
BMI, kg/m^2^	20.9 (2.3)	20.8 (2.5)	0.812
Clinical features			
Duration of disease, months	71.48 (60.1)	68.0 (65.2)	0.569
*Dyspepsia subtype*			0.992
PDS	143 (68.1%)	154 (68.1%)	
EPS	67 (31.9%)	72 (31.9%)	
Study design			
*Study site*			<0.001
Site A	134 (63.8%)	33 (14.6%)	
Site B	67 (31.9%)	91 (40.3%)	
Site C	9 (4.3%)	102 (45.1%)	
*Acupoint prescription*			0.035
Prescription A	61 (29.0%)	48 (21.2%)	
Prescription B	43 (20.5%)	66 (29.2%)	
Prescription C	57 (27.1%)	48 (21.2%)	
Prescription D	49 (23.3%)	64 (28.3%)	
Baseline symptoms measures			
SID	4.2 (1.3)	4.7 (1.8)	<0.001
Postprandial fullness	1.5 (0.7)	1.9 (0.8)	<0.001
Early satiety	1.1 (0.7)	1.3 (0.8)	<0.001
Epigastric pain	1.1 (0.8)	1.1 (0.9)	0.429
Epigastric burning	0.5 (0.7)	0.4 (0.7)	0.058
NDLQI	77.1 (9.1)	73.2 (10.7)	<0.001

Data are mean (SD) or *n* (%). Group A: junior acupuncturist treatment group. Group B: senior acupuncturist treatment group. BMI: body-mass index. PDS: postprandial distress syndrome. EPS: epigastric pain syndrome. SID: symptom index of dyspepsia. NDLQI: Nepean dyspepsia life quality index.

In addition, significant imbalances existed in study site distribution (*p* < 0.001) and acupoint prescription (*p* = 0.035). For baseline symptoms, no significant baseline symptom differences were observed in epigastric pain and epigastric burning (*p* > 0.05). However, the baseline symptoms showed significant differences in SID (4.2 ± 1.3 vs. 4.7 ± 1.8, *p* < 0.001), postprandial fullness (1.5 ± 0.7 vs. 1.9 ± 0.8, *p* < 0.001), early satiety (1.1 ± 0.7 vs. 1.3 ± 0.8, *p* < 0.001), and NDLQI (77.1 ± 9.1 vs. 73.2 ± 10.7, *p* < 0.001) (Table 3).

### Generalized estimating equations analysis of acupuncture outcomes

Since the study site, acupoint prescription, SID, postprandial fullness, early satiety and NDLQI differed between the two groups at baseline, these factors were included as covariates. After controlling for these baseline imbalances, the GEE analysis yielded the following results (Table 4).

**Table 4:** Adjusted analysis of acupuncture outcomes.

Outcome	Mean (SD)		Adjusted mean (SE)		Adjusted mean between-group difference (95% CI)	Adjusted *P* value
	Group A (*n* = 210)	Group B (*n* = 226)	Group A (*n* = 210)	Group B (*n* = 226)	Group A vs. Group B	
SID						
Baseline	4.2 (1.3)	4.7 (1.8)	4.3 (0.1)	4.5 (0.1)	−0.2 (−0.7,0.2)	1
Week 2 of treatment	2.8 (1.6)	3.2 (1.7)	2.9 (0.1)	3.0 (0.1)	−0.1 (−0.5,0.4)	1
Week 4 of treatment	2.3 (1.6)	2.5 (1.8)	2.5 (0.1)	2.3 (0.1)	0.2 (−0.3,0.7)	1
Week 4 of follow-up	2.4 (1.7)	2.2 (1.6)	2.6 (0.1)	2.0 (0.1)	0.6 (0.1,1.0)	0.007
Week 12 of follow-up	2.5 (1.7)	2.0 (1.5)	2.7 (0.1)	1.9 (0.1)	0.8 (0.3,1.3)	0.001
Postprandial fullness						
Baseline	1.5 (0.7)	1.9 (0.8)	1.6 (0.4)	1.8 (0.4)	−0.2 (−0.4,0.0)	0.435
Week 2 of treatment	1.1 (0.8)	1.4 (0.8)	1.3 (0.4)	1.2 (0.4)	0.1 (−0.2,0.3)	1
Week 4 of treatment	0.9 (0.7)	1.1 (0.8)	1.0 (0.4)	0.9 (0.4)	0.1 (−0.1,0.3)	1
Week 4 of follow-up	0.9 (0.7)	1.0 (0.7)	1.1 (0.4)	0.8 (0.4)	0.3 (0.1,0.5)	0.001
Week 12 of follow-up	1.0 (0.7)	0.9 (0.7)	1.1 (0.4)	0.8 (0.4)	0.4 (0.2,0.6)	<0.001
Early satiety						
Baseline	1.1 (0.7)	1.3 (0.8)	1.2 (0.4)	1.2 (0.4)	0.0 (−0.2,0.2)	1
Week 2 of treatment	0.7 (0.7)	0.9 (0.7)	0.8 (0.4)	0.8 (0.4)	0.0 (−0.2,0.2)	1
Week 4 of treatment	0.6 (0.7)	0.7 (0.7)	0.7 (0.4)	0.6 (0.4)	0.1 (−0.1,0.3)	1
Week 4 of follow-up	0.6 (0.7)	0.6 (0.6)	0.7 (0.4)	0.5 (0.4)	0.2 (0.0,0.4)	0.005
Week 12 of follow-up	0.7 (0.7)	0.6 (0.6)	0.8 (0.4)	0.5 (0.4)	0.3 (0.1,0.5)	<0.001
Epigastric pain						
Baseline	1.1 (0.8)	1.1 (0.9)	1.1 (0.5)	1.1 (0.5)	0.01 (−0.20,0.26)	1
Week 2 of treatment	0.7 (0.7)	0.7 (0.8)	0.7 (0.5)	0.7 (0.5)	0.03 (−0.23,0.28)	1
Week 4 of treatment	0.6 (0.7)	0.5 (0.7)	0.6 (0.5)	0.6 (0.5)	0.01 (−0.25,0.26)	1
Week 4 of follow-up	0.6 (0.7)	0.4 (0.6)	0.6 (0.5)	0.5 (0.5)	0.12 (−0.13,0.38)	1
Week 12 of follow-up	0.6 (0.7)	0.4 (0.6)	0.6 (0.5)	0.4 (0.5)	0.18 (−0.07,0.44)	0.813
Epigastric burning						
Baseline	0.5 (0.7)	0.4 (0.6)	0.40 (0.04)	0.43 (0.04)	−0.04 (−0.22,0.15)	1
Week 2 of treatment	0.3 (0.5)	0.3 (0.5)	0.20 (0.04)	0.34 (0.04)	−0.14 (−0.32,0.04)	0.598
Week 4 of treatment	0.3 (0.5)	0.2 (0.5)	0.20 (0.04)	0.28 (0.04)	−0.08 (−0.26,0.11)	1
Week 4 of follow-up	0.2 (0.5)	0.2 (0.4)	0.16 (0.04)	0.24 (0.04)	−0.08 (−0.26,0.10)	1
Week 12 of follow-up	0.2 (0.5)	0.2 (0.4)	0.17 (0.04)	0.23 (0.04)	−0.07 (−0.25,0.12)	1
NDLQI						
Baseline	77.1 (9.1)	73.2 (10.7)	75.8 (0.6)	74.5 (0.6)	1.3 (−1.5,4.1)	1
Week 2 of treatment	85.6 (9.5)	81.5 (10.2)	84.2 (0.6)	82.7 (0.6)	1.5 (−1.3,4.3)	1
Week 4 of treatment	87.9 (9.2)	85.4 (10.4)	86.6 (0.6)	86.7 (0.6)	−0.1 (−2.8,2.7)	1
Week 4 of follow-up	87.8 (9.2)	86.9 (9.6)	86.5 (0.6)	88.2 (0.6)	−1.7 (−4.5,1.1)	1
Week 12 of follow-up	87.5 (8.5)	87.8 (9.5)	86.2 (0.6)	89.0 (0.6)	−2.9 (−5.7,-0.1)	0.041

Group A: junior acupuncturist treatment group. Group B: senior acupuncturist treatment group. SID: symptom index of dyspepsia. NDLQI, Nepean dyspepsia life quality index.

**Figure 2:**
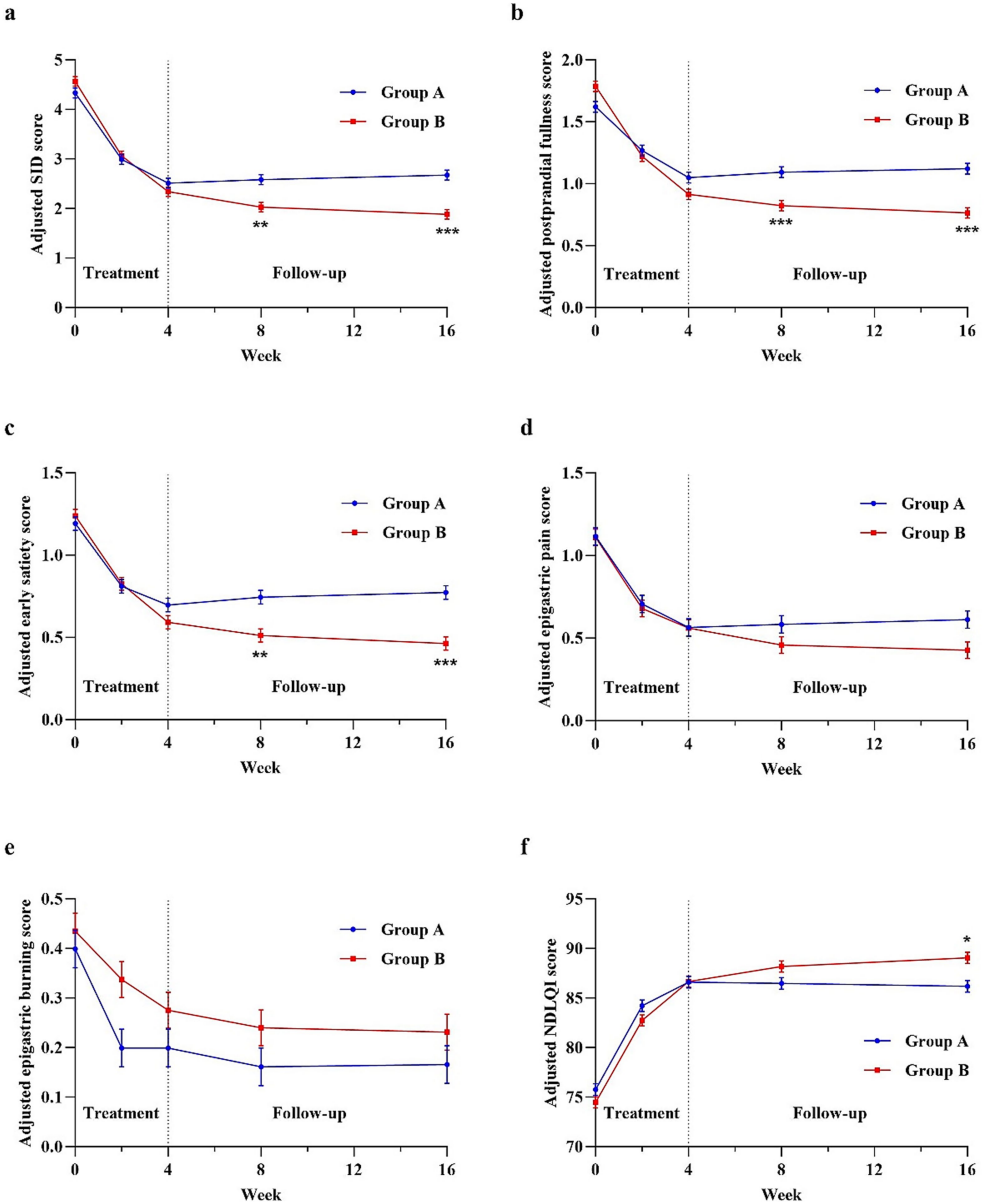
Adjusted analysis of outcomes for two group FD patients. **(a)–(f)** show the adjusted scores for SID, postprandial fullness, early satiety, epigastric pain, epigastric burning, and NDLQI, respectively, over the 16‑week period including baseline, 4 weeks of treatment, and 12 weeks of follow‑up. Group A: junior acupuncturist treatment group. Group B: senior acupuncturist treatment group. SID, symptom index of dyspepsia; NDLQI, Nepean dyspepsia life quality index. Comparison between two groups: *p < 0.05; **p < 0.01; ***p < 0.001.

For overall dyspeptic symptoms, patients in Group B showed a significantly lower SID score than Group A at the 4-week follow-up (mean difference 0.6, 95% CI: 0.1 to 1.0, *p* = 0.007). The difference widened further at the 12-week follow-up (mean difference 0.8, 95% CI: 0.3 to 1.3, *p* = 0.001) (Figure 2a).

For the four cardinal symptoms, patients in Group B experienced significantly greater improvement in postprandial fullness and early satiety than Group A at both the 4-week and 12-week follow-ups (*p* < 0.01) (Figures 2b,c). There were no statistically significant differences between the two groups in terms of epigastric pain or burning at any time point (*p* > 0.05) (Figures 2d,e).

Regarding quality of life for FD patients, patients in Group B had a significantly higher NDLQI score at the 12-week follow-up compared to Group A (−2.9, 95% CI: −5.7 to −0.1, *p* = 0.041) (Figure 2f).

### Exploratory analysis outcomes of subgroups

According to the above statistical method, we conducted an exploratory analysis of the two subtypes of FD (details were provided in the Supplementary material). Based on the PDS-domain and EPS-domain groups, the results showed similar trends to those observed in the overall FD patient population. Notably, the therapeutic benefit of senior acupuncturists on epigastric pain was evident in both subgroups. Compared with Group A (treated by junior acupuncturists), patients in Group B (treated by senior acupuncturists) showed significant improvement in epigastric pain at the end of the 12-week follow-up (mean difference [A–B]: PDS-domain group: mean difference 0.3, 95% CI: 0.0 to 0.5, *p* = 0.006; EPS-domain group: mean difference 0.3, 95% CI: 0.1 to 0.5, *p* = 0.002).

## Discussion

Based on the results of this exploratory secondary analysis based on a 5-year administrative cutoff for professional title classification, during the follow-up period, FD patients in the senior acupuncturist group experienced more symptom relief than those in the junior acupuncturist group, and similar results were observed in the symptom-domain-based subgroup analysis. Some large-scale, multicenter clinical studies have also shown that acupuncture effects persist during the follow-up period (16, 23). However, at the end of the 4-week treatment, no difference was found between the two groups, which may be attributable to the acupuncture effects in both groups reaching a saturation point. Regarding the observed association between acupuncturists’ years of practice and acupuncture efficacy, we propose the following hypotheses based on clinical observations and literature evidence:

Firstly, acupuncturists with different experience levels may differ in their acupuncture manipulation. According to clinical observations and literature evidence, acupuncturists’ practical experience may be externally reflected in aspects of acupuncture manipulation such as acupoint localization and the ability to induce *deqi* (11, 12, 24, 25). For acupuncture manipulation, the direction, angle, and depth of needling are usually considered as important factors affecting acupuncture efficacy (26); therefore, in acupuncture clinical teaching and research, acupuncturists are generally required to follow the requirements for standardization of acupuncture manipulation. However, even for established standardized acupuncture procedures, different acupuncturists may differ in their understanding and execution. For example, Liu et al. (27) used an acupuncture manipulation information acquisition and analysis system to collect data on 6 acupuncture manipulations (all of which have established operating paradigms in textbooks) performed by 10 professional acupuncturists, divided into two types: teacher-type and clinical-type. The acquired parameters included the amplitude, frequency, and duration of the acupuncture manipulations. Their results showed significant differences in all parameters of acupuncture manipulation between the two types of acupuncturists, indicating that different acupuncturists do not fully understand and execute standardized acupuncture manipulations in a completely consistent manner. In addition, neuroscientific evidence has demonstrated that practice experience may influence the neuroplasticity of acupuncturists. A series of studies using functional magnetic resonance imaging (fMRI) compared spontaneous brain activity during rest between professional acupuncturists and non-acupuncturists (28–30). The results showed that the professional acupuncturists exhibited higher local synchronization in brain regions related to sensorimotor and emotion regulation, and this synchronization was correlated with their acupuncture experience level and years of practice. These studies suggested that specialized acupuncture training can promote the reorganization and enhancement of local neural activity in brain regions, reflecting the resting-state characteristics of experience-dependent neuroplasticity. Indeed, not only in acupuncture, but also in other non-pharmacological interventions, such as surgery (31), psychotherapy (32) and physical therapy (33), the operator’s experience level and manipulation level are often considered key factors influencing treatment efficacy (34–36). Therefore, we speculate that there may be subtle differences in acupuncture manipulation among acupuncturists with different experience levels, and such differences may be one of the factors affecting the therapeutic effect of acupuncture. Moreover, in this study, senior acupuncturists had a high proportion of advanced degrees. Educational background may also have a potential influence, as studies have shown that more systematic acupuncture training is positively associated with improvements in acupuncturists’ theoretical and practical abilities (37).

Secondly, beyond acupuncture manipulation, the differences in acupuncture efficacy between the two groups of acupuncturists may also be related to doctor-patient interaction, which can be summarized as the non-specific effects of acupuncture—also known as the placebo effect (13, 38). In acupuncture, a therapy that places particular emphasis on doctor-patient interaction, patients may pay more attention to elements such as the acupuncturist’s appearance, therapeutic ritual, doctor-patient relationship, and the acupuncturist’s assessment and observation (36, 39–41). These elements may influence patients’ confidence and expectations regarding acupuncture treatment (42–44). Specifically, for example, in an RCT of acupuncture for knee osteoarthritis (36), acupuncturists were trained to interact in one of two communication styles: high expectation or neutral expectation. The results showed that in the high-expectation communication style, patients had higher satisfaction with the acupuncturist, and it also had a positive impact on pain relief. This suggests that the analgesic effect of acupuncture may be partially mediated by placebo effects related to acupuncturist behavior. Furthermore, some studies indicate that higher-quality acupuncturists tend to produce more significant placebo effects in clinical practice (3, 9, 10). Therefore, we speculate that patients may receive more positive feedback in their interactions with senior acupuncturists, and such positive doctor–patient interaction is correlated with the improvement of FD symptoms. Neuroimaging studies further explain the neural mechanisms underlying positive doctor-patient interaction (45–48). A study based on event-related potentials suggested that positive doctor-patient relationships led to increased patient attention and rapport, as well as larger P2 amplitudes (49). Furthermore, another fMRI study found that better doctor-patient communication led to greater interaction between the two parties and increased inter-brain activity in the temporoparietal junction during treatment (50). These studies are considered the neural basis through which doctor-patient communication influences the non-specific therapeutic effects of acupuncture. Given the importance of doctor-patient communication in improving acupuncture efficacy described above, the causal relationship between doctor-patient communication and acupuncture efficacy, as well as the underlying neural mechanisms, is a scientific issue worthy of in-depth investigation in the future.

Based on the outcomes in this study and the above speculation, we offer the following recommendations as a reference for future clinical practice and research on acupuncture. On the one hand, acupuncture practice requires a focus on continuing education and standardized training and assessment for practitioners, such as regularly specialized training in acupuncture manipulation, and doctor-patient communication (8). On the other hand, acupuncture clinical trials require more rigorous control over the operator’s influence on treatment effects. Current acupuncture clinical studies rarely report the range of acupuncturists’ years of practice, with most reporting only the minimum. Future acupuncture clinical trials should report in greater detail for acupuncturists’ years of practice across different study centers, using randomization to stratify by acupuncturist experience level in order to reduce baseline imbalance.

This study has several important limitations. Firstly, the findings of this study are based on a non-randomized secondary grouping using a 5-year cutoff as an administrative division, yielding exploratory associations that cannot directly assess differences among acupuncturists in acupuncture manipulation, clinical communication, or other relevant skills, such as *deqi* induction, acupoint localization, or patient-practitioner communication. Therefore, all statements in the above discussion regarding acupuncture manipulation, patient-practitioner interaction, and other factors used to explain the findings of this study are speculative. Furthermore, this study did not conduct a continuous variable analysis based on this grouping threshold, and whether a linear relationship exists between acupuncturist experience level and acupuncture efficacy for FD remains uncertain. Secondly, although baseline items with significant between-group differences were included as covariates in the analysis, given that most patients in the senior acupuncturist group had more severe baseline symptoms under non-randomized conditions, the influence of regression to the mean bias in the observed outcomes of this group cannot be ruled out. Thirdly, given the small sample size of acupuncturists (*n* = 12), cluster analysis was not performed to avoid unstable estimates; therefore, the potential impact of individual differences among acupuncturists on treatment effects could not be assessed. At last, further subgroup analysis based on specific characteristics of acupuncturists and FD patients would have led to extreme sample size imbalances between groups. Consequently, no additional sensitivity analyses were conducted, and residual confounding factors cannot be completely excluded. For example, the two acupuncturist groups were imbalanced in sex distribution, so the results may be influenced by non-specific factors such as patient expectations.

## Conclusion

Based on the 5-year administrative classification criterion, this exploratory secondary analysis found an association between acupuncturists’ experience level and symptom relief in FD patients during follow-up. These findings offer a preliminary reference for understanding the effect of acupuncturists’ years of practice on acupuncture efficacy for FD. However, due to the inherent limitations of secondary analysis, prospective studies are needed to further confirm these findings.

## Data Availability

The raw data supporting the conclusions of this article will be made available by the authors, without undue reservation.
