# Supplementary material for: The impact of acupuncturists’ experience level on the efficacy of acupuncture for functional dyspepsia: an exploratory secondary analysis of a randomized clinical trial
Source: Front Med (Lausanne). 2026 Jun 11;13:1834168. doi: 10.3389/fmed.2026.1834168 (PMC13293913; doi:10.3389/fmed.2026.1834168)
Supplement: Supplementary file 1 [file Supplementary_file_1.docx]

**Supplementary material**

Contents

[Results for the PDS-Domain Group 2](#_Toc227058263)

[Characteristics of Acupuncturists Involved in Treating the PDS-Domain Group 2](#_Toc227058264)

[Baseline characteristics for PDS-Domain Group patients 3](#_Toc227058265)

[Generalized Estimating Equation Analysis for PDS-Domain Group Patients 5](#_Toc227058266)

[Results for the EPS-Domain Group 7](#_Toc227058267)

[Characteristics of Acupuncturists Involved in Treating the EPS -Domain Group 7](#_Toc227058268)

[Baseline characteristics for EPS-Domain Group patients 8](#_Toc227058269)

[Generalized Estimating Equation Analysis for EPS-Domain Group Patients 10](#_Toc227058270)

[Detailed Acupuncture Prescriptions and Procedures 12](#_Toc227058271)

[Acupoint prescriptions 12](#_Toc227058272)

[Acupuncture procedures 12](#_Toc227058273)

# Results for the PDS-Domain Group

## Characteristics of Acupuncturists Involved in Treating the PDS-Domain Group

| **S1 Table. Characteristics of Acupuncturists Involved in Treating the PDS-Domain Group** | | | |
| --- | --- | --- | --- |
| **Characteristic** | **Junior acupuncturist (n=5)** | **Senior acupuncturist (n=7)** | ***P* Value** |
| Age, years | 24.8 (0.84) | 31.9 (5.6) | 0.004 |
| Sex |  |  | 0.205 |
| Female | 5 (100%) | 4 (57.1%) |  |
| Male | 0 (0.0%) | 3 (42.9%) |  |
| Acupuncture practice, years | 2.4 (0.5) | 9.4 (5.3) | 0.004 |
| Patient treated, numbers | 39.8 (53.7) | 30.6 (32.9) | 0.684 |
| Education level |  |  | 0.023 |
| Bachelor's degree | 5 (100%) | 1 (14.3%) |  |
| Master's degree | 0 (0.0%) | 4 (57.1%) |  |
| Doctoral degree | 0 (0.0%) | 2 (28.6%) |  |
| Study site |  |  | 0.886 |
| Site A | 1 (20.0%) | 2 (28.6%) |  |
| Site B | 3 (60.0%) | 3 (42.9%) |  |
| Site C | 1 (20.0%) | 2 (28.6%) |  |

Data are mean (SD) or n (%).

## Baseline characteristics for PDS-Domain Group patients


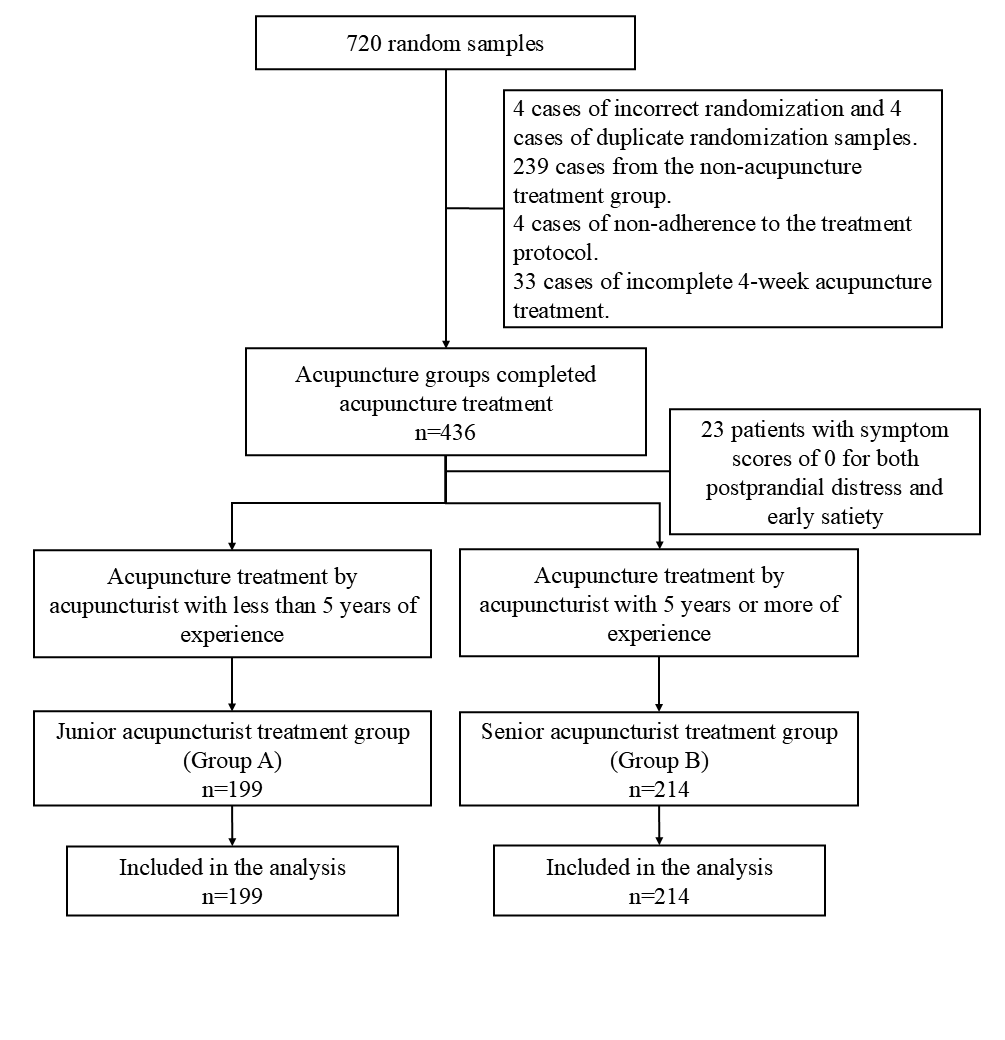


**S1 Figure. Trial flow chart for PDS-domain group patients.**

| **S2 Table. Baseline characteristics and outcome measures for PDS-** **Domain Group patients** | | | |
| --- | --- | --- | --- |
| **Characteristic** | **Group A** | **Group B** | ***P* Value** |
|  | **(n=199)** | **(n=214)** |  |
| **Demographics** | | | |
| Age, years | 37.9 (13.8) | 36.7 (13.3) | 0.363 |
| Sex |  |  | 0.302 |
| Female | 145 (72.9%) | 146 (68.2%) |  |
| Male | 54 (27.1%) | 68 (31.8%) |  |
| BMI, kg/m^2^ | 20.9 (2.4) | 20.9 (2.5) | 0.816 |
| **Clinical Features** | | | |
| Duration of disease, months | 72.4 (61.2) | 68.9 (66.2) | 0.575 |
| **Study Design** | | | |
| Study site |  |  | < 0.001 |
| Site A | 123 (61.8%) | 29 (13.6%) |  |
| Site B | 67 (33.7%) | 87 (40.7%) |  |
| Site C | 9 (4.5%) | 98 (45.8%) |  |
| Acupoint prescription |  |  | 0.016 |
| Prescription A | 58 (29.1%) | 45 (21.0%) |  |
| Prescription B | 39 (19.6%) | 61 (28.5%) |  |
| Prescription C | 56 (28.1%) | 44 (20.6%) |  |
| Prescription D | 46 (23.1%) | 64 (29.9%) |  |
| **Baseline Symptoms Measures** | | | |
| SID | 4.3 (1.3) | 4.8 (1.8) | < 0.001 |
| Postprandial fullness | 1.5 (0.6) | 2.0 (0.7) | < 0.001 |
| Early satiety | 1.1 (0.7) | 1.4 (0.8) | < 0.001 |
| Epigastric pain | 1.1 (0.8) | 1.1 (0.9) | 0.358 |
| Epigastric burning | 0.5 (0.7) | 0.4 (0.7) | 0.047 |
| NDLQI | 77.0 (9.3) | 72.9 (10.6) | < 0.001 |

Data are mean (SD) or n (%). Group A: junior acupuncturist treatment group. Group B: senior acupuncturist treatment group. SID: symptom index of dyspepsia. NDLQI: Nepean dyspepsia life quality index.

## Generalized Estimating Equation Analysis for PDS-Domain Group Patients

| **S3 Table. Adjusted Analysis of Outcomes for PDS -Domain Group Patients** | | | | | | |
| --- | --- | --- | --- | --- | --- | --- |
| **Outcome** | **Mean (SD)** | | **Adjusted Mean (SE)** | | **Adjusted mean between-group difference (95% CI)** | **Adjusted *P* value** |
|  | **Group A** | **Group B** | **Group A** | **Group B** | **Group A vs. Group B** |  |
|  | **(n=199)** | **(n=214)** | **(n=199)** | **(n=214)** |  |  |
| **SID** |  |  |  |  |  |  |
| Baseline | 4.3 (1.3) | 4.8 (1.8) | 4.4 (0.1) | 4.7 (0.1) | -0.3 (-0.7,0.3) | 1 |
| Week 2 of treatment | 2.9 (1.6) | 3.3 (1.7) | 3.1 (0.1) | 3.1 (0.1) | 0.0 (-0.5,0.4) | 1 |
| Week 4 of treatment | 2.4 (1.7) | 2.5 (1.8) | 2.6 (0.1) | 2.4 (0.1) | 0.2 (-0.3,0.7) | 1 |
| Week 4 of follow-up | 2.5 (1.7) | 2.2 (1.6) | 2.6 (0.1) | 2.0 (0.1) | 0.6 (0.1,1.1) | 0.004 |
| Week 12 of follow-up | 2.5 (1.7) | 2.1 (1.5) | 2.7 (0.1) | 1.9 (0.1) | 0.8 (0.3,1.3) | < 0.001 |
| **Postprandial fullness** |  |  |  |  |  |  |
| Baseline | 1.5 (0.6) | 2.0 (0.7) | 1.7 (0.04) | 1.9 (0.04) | -0.2 (-0.4,0.1) | 0.374 |
| Week 2 of treatment | 1.2 (0.8) | 1.4 (0.7) | 1.3 (0.04) | 1.3 (0.04) | 0.0 (-0.2,0.3) | 1 |
| Week 4 of treatment | 0.9 (0.7) | 1.1 (0.7) | 1.1 (0.04) | 1.0 (0.04) | 0.1 (-0.1,0.3) | 1 |
| Week 4 of follow-up | 1.0 (0.7) | 1.0 (0.7) | 1.1 (0.04) | 0.8 (0.04) | 0.3 (0.1,0.5) | 0.002 |
| Week 12 of follow-up | 1.0 (0.7) | 0.9 (0.7) | 1.1 (0.04) | 0.7 (0.04) | 0.4 (0.1,0.6) | < 0.001 |
| **Early satiety** |  |  |  |  |  |  |
| Baseline | 1.1 (0.7) | 1.4 (0.8) | 1.3 (0.04) | 1.3 (0.04) | 0.0 (-0.3,0.2) | 1 |
| Week 2 of treatment | 0.7 (0.7) | 1.0 (0.7) | 0.8 (0.04) | 0.8 (0.04) | 0.0 (-0.2,0.2) | 1 |
| Week 4 of treatment | 0.6 (0.7) | 0.7 (0.7) | 0.7 (0.04) | 0.6 (0.04) | 0.1 (-0.1,0.3) | 1 |
| Week 4 of follow-up | 0.6 (0.7) | 0.6 (0.6) | 0.8 (0.04) | 0.5 (0.04) | 0.3 (0.0,0.4) | 0.015 |
| Week 12 of follow-up | 0.7 (0.7) | 0.6 (0.6) | 0.8 (0.04) | 0.5 (0.04) | 0.3 (0.1,0.5) | < 0.001 |
| **Epigastric pain** |  |  |  |  |  |  |
| Baseline | 1.1 (0.8) | 1.0 (1.0) | 1.1 (0.05) | 1.0 (0.05) | 0.1 (-0.2,0.3) | 1 |
| Week 2 of treatment | 0.7 (0.7) | 0.6 (0.8) | 0.7 (0.05) | 0.6 (0.05) | 0.1 (-0.1,0.3) | 1 |
| Week 4 of treatment | 0.6 (0.7) | 0.5 (0.7) | 0.6 (0.05) | 0.5 (0.05) | 0.1 (-0.1,0.3) | 1 |
| Week 4 of follow-up | 0.6 (0.7) | 0.4 (0.6) | 0.6 (0.05) | 0.4 (0.05) | 0.2 (-0.1,0.4) | 0.149 |
| Week 12 of follow-up | 0.6 (0.7) | 0.4 (0.6) | 0.6 (0.05) | 0.3 (0.05) | 0.3 (0.0,0.5) | 0.006 |
| **Epigastric burning** |  |  |  |  |  |  |
| Baseline | 0.5 (0.7) | 0.4 (0.7) | 0.42 (0.03) | 0.38 (0.03) | 0.04 (-0.09,0.17) | 1 |
| Week 2 of treatment | 0.3 (0.5) | 0.3 (0.5) | 0.24 (0.03) | 0.28 (0.03) | -0.04 (-0.16,0.09) | 1 |
| Week 4 of treatment | 0.3 (0.5) | 0.2 (0.4) | 0.24 (0.03) | 0.22 (0.03) | 0.02 (-0.11,0.14) | 1 |
| Week 4 of follow-up | 0.2 (0.5) | 0.2 (0.4) | 0.20 (0.03) | 0.18 (0.03) | 0.02 (-0.11,0.15) | 1 |
| Week 12 of follow-up | 0.2 (0.5) | 0.1 (0.4) | 0.20 (0.03) | 0.17 (0.03) | 0.03 (0.09,0.16) | 1 |
| **NDLQI** |  |  |  |  |  |  |
| Baseline | 77.0 (9.3) | 73 (10.6) | 75.7 (0.6) | 74.2 (0.6) | 1.5 (-1.1,4.2) | 1 |
| Week 2 of treatment | 85.5 (9.6) | 81.5 (10.1) | 84.2 (0.6) | 82.7 (0.6) | 1.5 (-1.2,4.1) | 1 |
| Week 4 of treatment | 87.8 (9.4) | 85.5 (10.4) | 86.5 (0.6) | 86.7 (0.6) | -0.1 (-2.7,2.5) | 1 |
| Week 4 of follow-up | 87.7 (9.3) | 86.9 (9.6) | 86.4 (0.6) | 88.1 (0.6) | -1.7 (-4.3,0.9) | 1 |
| Week 12 of follow-up | 87.4 (8.7) | 87.6 (9.5) | 86.1 (0.6) | 88.8 (0.6) | -2.6 (-5.3,-0.1) | 0.045 |

Group A: junior acupuncturist treatment group. Group B: senior acupuncturist treatment group. SID: symptom index of dyspepsia. NDLQI: Nepean dyspepsia life quality index.


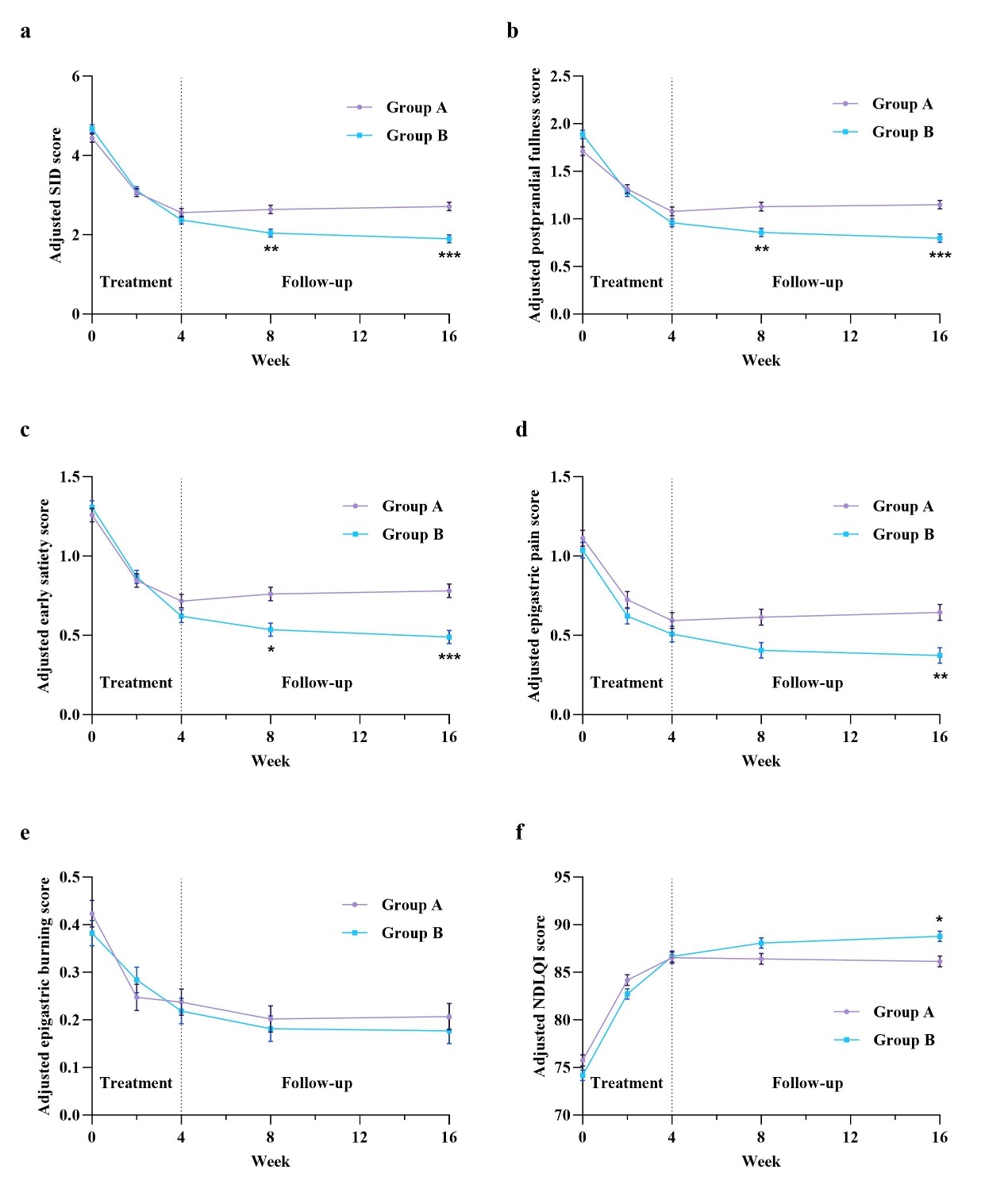


**S2 Figure. Adjusted Analysis of Outcomes for PDS-Domain Group Patients**

(a)–(f) show the adjusted scores for SID, postprandial fullness, early satiety, epigastric pain, epigastric burning, and NDLQI, respectively, over the 16‑week period including baseline, 4 weeks of treatment, and 12 weeks of follow‑up. Group A: junior acupuncturist treatment group. Group B: senior acupuncturist treatment group. SID: symptom index of dyspepsia. NDLQI: Nepean dyspepsia life quality index. Comparison between two groups: **^*^***P* < 0.05; **^**^***P* < 0.01; **^***^***P* < 0.001.

# Results for the EPS-Domain Group

## Characteristics of Acupuncturists Involved in Treating the EPS -Domain Group

| **S4 Table. Characteristics of Acupuncturists Involved in Treating the EPS-Domain Group** | | | |
| --- | --- | --- | --- |
| **Characteristic** | **Junior acupuncturist (n=5)** | **Senior acupuncturist (n=7)** | ***P* Valu*e*** |
| Age, years | 24.8 (0.84) | 31.9 (5.6) | 0.004 |
| Sex |  |  | 0.205 |
| Female | 5 (100%) | 4 (57.1%) |  |
| Male | 0 (0.0%) | 3 (42.9%) |  |
| Acupuncture practice, years | 2.4 (0.5) | 9.4 (5.3) | 0.004 |
| Patient treated, numbers | 34.6 (50.6) | 22.9 (23.9) | 0.625 |
| Education level |  |  | 0.023 |
| Bachelor's degree | 5 (100%) | 1 (14.3%) |  |
| Master's degree | 0 (0.0%) | 4 (57.1%) |  |
| Doctoral degree | 0 (0.0%) | 2 (28.6%) |  |
| Study site |  |  | 0.886 |
| Site A | 1 (20.0%) | 2 (28.6%) |  |
| Site B | 3 (60.0%) | 3 (42.9%) |  |
| Site C | 1 (20.0%) | 2 (28.6%) |  |

Data are mean (SD) or n (%).

## Baseline characteristics for EPS-Domain Group patients


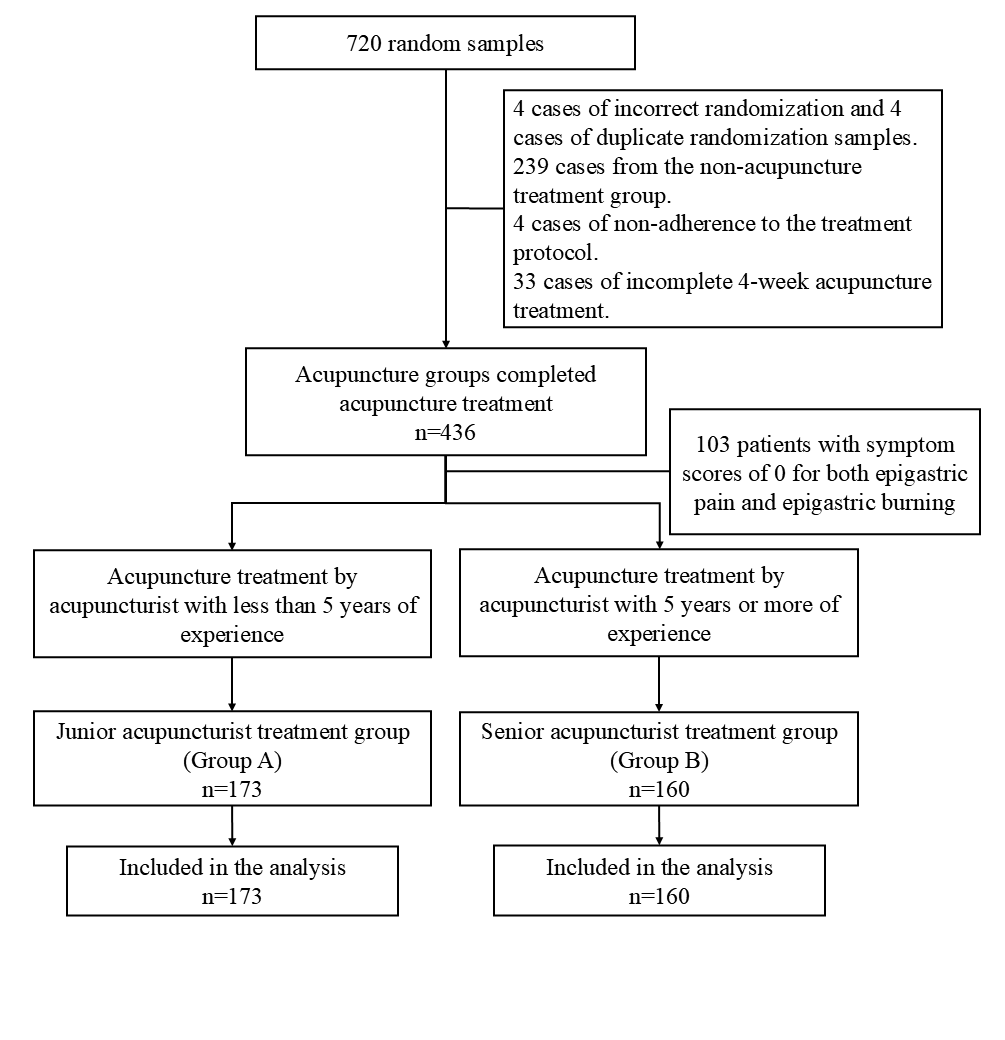


**S3 Figure. Trial flow chart for EPS-domain group patients.**

| **Table S5. Baseline characteristics and outcome measures for EPS-Domain Group patients** | | | |
| --- | --- | --- | --- |
| **Characteristic** | **Group A** | **Group B** | ***P* Value** |
|  | **(n=173)** | **(n=160)** |  |
| **Demographics** | | | |
| Age, years | 39.2 (13.8) | 36.3 (13.5) | 0.059 |
| Sex |  |  | 0.122 |
| Female | 134 (77.5%) | 112 (70.0%) |  |
| Male | 39 (22.5%) | 48 (30.0%) |  |
| BMI, kg/m^2^ | 21 (2.4) | 20.9 (2.7) | 0.786 |
| **Clinical Features** | | | |
| Duration of disease, months | 75.5 (57.8) | 71.2 (64.7) | 0.521 |
| **Study Design** | | | |
| Study site |  |  | < 0.001 |
| Site A | 118 (68.2%) | 22 (13.8%) |  |
| Site B | 50 (28.9%) | 68 (42.5%) |  |
| Site C | 5 (2.9%) | 70 (43.8%) |  |
| Acupoint prescription |  |  | 0.099 |
| Prescription A | 51 (29.5%) | 32 (20.0%) |  |
| Prescription B | 34 (19.7%) | 45 (28.1%) |  |
| Prescription C | 45 (26.0%) | 37 (23.1%) |  |
| Prescription D | 43 (24.9%) | 46 (28.7%) |  |
| **Baseline Symptoms Measures** | | | |
| SID | 4.4 (1.3) | 5.2 (1.8) | < 0.001 |
| Postprandial fullness | 1.4 (0.7) | 1.9 (0.9) | < 0.001 |
| Early satiety | 1.0 (0.7) | 1.3 (0.9) | 0.016 |
| Epigastric pain | 1.4 (0.6) | 1.5 (0.8) | 0.039 |
| Epigastric burning | 0.6 (0.7) | 0.5 (0.7) | 0.409 |
| NDLQI | 76.6 (8.9) | 73.1 (9.7) | 0.001 |

Data are mean (SD) or n (%). Group A: junior acupuncturist treatment group. Group B: senior acupuncturist treatment group. SID: symptom index of dyspepsia. NDLQI: Nepean dyspepsia life quality index.

## Generalized Estimating Equation Analysis for EPS-Domain Group Patients

| **Table S6. Adjusted Analysis of Outcomes for EPS-Domain Group Patients** | | | | | | |
| --- | --- | --- | --- | --- | --- | --- |
| **Outcome** | **Mean (SD)** | | **Adjusted Mean (SE)** | | **Adjusted mean between-group difference (95% CI)** | **Adjusted *P* value** |
|  | **Group A** | **Group B** | **Group A** | **Group B** | **Group A vs. Group B** |  |
|  | **(n=173)** | **(n=160)** | **(n=173)** | **(n=160)** |  |  |
| **SID** |  |  |  |  |  |  |
| Baseline | 4.4 (1.3) | 5.2 (1.8) | 4.6 (0.1) | 4.9 (0.1) | -0.3 (-0.9,0.3) | 1 |
| Week 2 of treatment | 2.9 (1.7) | 3.5 (1.7) | 3.1 (0.1) | 3.3 (0.1) | -0.2 (-0.8,0.4) | 1 |
| Week 4 of treatment | 2.5 (1.7) | 2.8 (1.9) | 2.7 (0.1) | 2.5 (0.1) | 0.2 (-0.4,0.8) | 1 |
| Week 4 of follow-up | 2.5 (1.8) | 2.3 (1.7) | 2.7 (0.1) | 2.1 (0.1) | 0.7 (0.0,1.3) | 0.02 |
| Week 12 of follow-up | 2.6 (1.7) | 2.2 (1.6) | 2.9 (0.1) | 2.0 (0.1) | 0.9 (0.3,1.5) | < 0.001 |
| **Postprandial fullness** |  |  |  |  |  |  |
| Baseline | 1.4 (0.7) | 1.9 (0.9) | 1.5 (0.1) | 1.7 (0.1) | -0.2 (-0.4,0.1) | 1 |
| Week 2 of treatment | 1.0 (0.8) | 1.3 (0.8) | 1.2 (0.1) | 1.2 (0.1) | 0.0 (-0.2,0.3) | 1 |
| Week 4 of treatment | 0.9 (0.7) | 1.1 (0.8) | 1.0 (0.1) | 0.9 (0.1) | 0.1 (-0.1,0.4) | 1 |
| Week 4 of follow-up | 0.9 (0.7) | 0.9 (0.7) | 1.1 (0.1) | 0.7 (0.1) | 0.3 (0.1,0.6) | 0.001 |
| Week 12 of follow-up | 0.9 (0.7) | 0.9 (0.7) | 1.1 (0.1) | 0.7 (0.1) | 0.4 (0.1,0.6) | < 0.001 |
| **Early satiety** |  |  |  |  |  |  |
| Baseline | 1.0 (0.7) | 1.3 (0.9) | 1.1 (0.05) | 1.1 (0.05) | 0.0 (-0.3,0.2) | 1 |
| Week 2 of treatment | 0.6 (0.7) | 0.9 (0.7) | 0.7 (0.05) | 0.8 (0.05) | -0.1 (-0.3,0.1) | 1 |
| Week 4 of treatment | 0.6 (0.7) | 0.7 (0.7) | 0.7 (0.05) | 0.6 (0.05) | 0.1 (-0.2,0.3) | 1 |
| Week 4 of follow-up | 0.6 (0.7) | 0.6 (0.6) | 0.7 (0.05) | 0.5 (0.05) | 0.2 (0.0,0.4) | 0.177 |
| Week 12 of follow-up | 0.6 (0.7) | 0.5 (0.6) | 0.7 (0.05) | 0.4 (0.05) | 0.3 (0.1,0.5) | 0.001 |
| **Epigastric pain** |  |  |  |  |  |  |
| Baseline | 1.4 (0.6) | 1.5 (0.8) | 1.4 (0.05) | 1.5 (0.05) | -0.1 (-0.3,0.2) | 1 |
| Week 2 of treatment | 0.9 (0.7) | 0.9 (0.8) | 0.9 (0.05) | 0.9 (0.05) | 0.1 (-0.2,0.3) | 1 |
| Week 4 of treatment | 0.7 (0.7) | 0.7 (0.7) | 0.7 (0.05) | 0.7 (0.05) | 0.1 (-0.2,0.3) | 1 |
| Week 4 of follow-up | 0.7 (0.7) | 0.6 (0.6) | 0.8 (0.05) | 0.6 (0.05) | 0.2 (0.0,0.4) | 0.133 |
| Week 12 of follow-up | 0.8 (0.7) | 0.6 (0.7) | 0.8 (0.05) | 0.5 (0.05) | 0.3 (0.1,0.5) | 0.002 |
| **Epigastric burning** |  |  |  |  |  |  |
| Baseline | 0.6 (0.7) | 0.5 (0.7) | 0.5 (0.05) | 0.6 (0.05) | -0.1 (-0.3,0.1) | 1 |
| Week 2 of treatment | 0.3 (0.5) | 0.4 (0.6) | 0.2 (0.05) | 0.4 (0.05) | -0.2 (-0.5,0.0) | 1 |
| Week 4 of treatment | 0.3 (0.6) | 0.3 (0.5) | 0.2 (0.05) | 0.4 (0.05) | -0.1 (-0.4,0.1) | 1 |
| Week 4 of follow-up | 0.3 (0.6) | 0.2 (0.5) | 0.2 (0.05) | 0.3 (0.05) | -0.1 (-0.3,0.1) | 1 |
| Week 12 of follow-up | 0.3 (0.6) | 0.2 (0.4) | 0.2 (0.05) | 0.3 (0.05) | -0.1 (-0.3,0.1) | 1 |
| **NDLQI** |  |  |  |  |  |  |
| Baseline | 76.6 (8.9) | 73.1 (9.7) | 75.6 (0.6) | 74.2 (0.7) | 1.4 (-1.6,4.4) | 1 |
| Week 2 of treatment | 85.2 (9.8) | 81.1 (10) | 84.2 (0.6) | 82.2 (0.7) | 2.0 (-1.0,5.0) | 1 |
| Week 4 of treatment | 87.7 (9.7) | 84.9 (10.6) | 86.6 (0.6) | 86.0 (0.7) | 0.7 (-2.3,3.6) | 1 |
| Week 4 of follow-up | 87.7 (9.6) | 86.6 (9.5) | 86.6 (0.6) | 87.7 (0.7) | -1.1 (-4.1,1.9) | 1 |
| Week 12 of follow-up | 87.3 (8.8) | 87.5 (9.6) | 86.3 (0.6) | 88.6 (0.7) | -2.2 (-5.2,0.8) | 0.687 |

Group A: junior acupuncturist treatment group. Group B: senior acupuncturist treatment group. SID: symptom index of dyspepsia. NDLQI: Nepean dyspepsia life quality index.


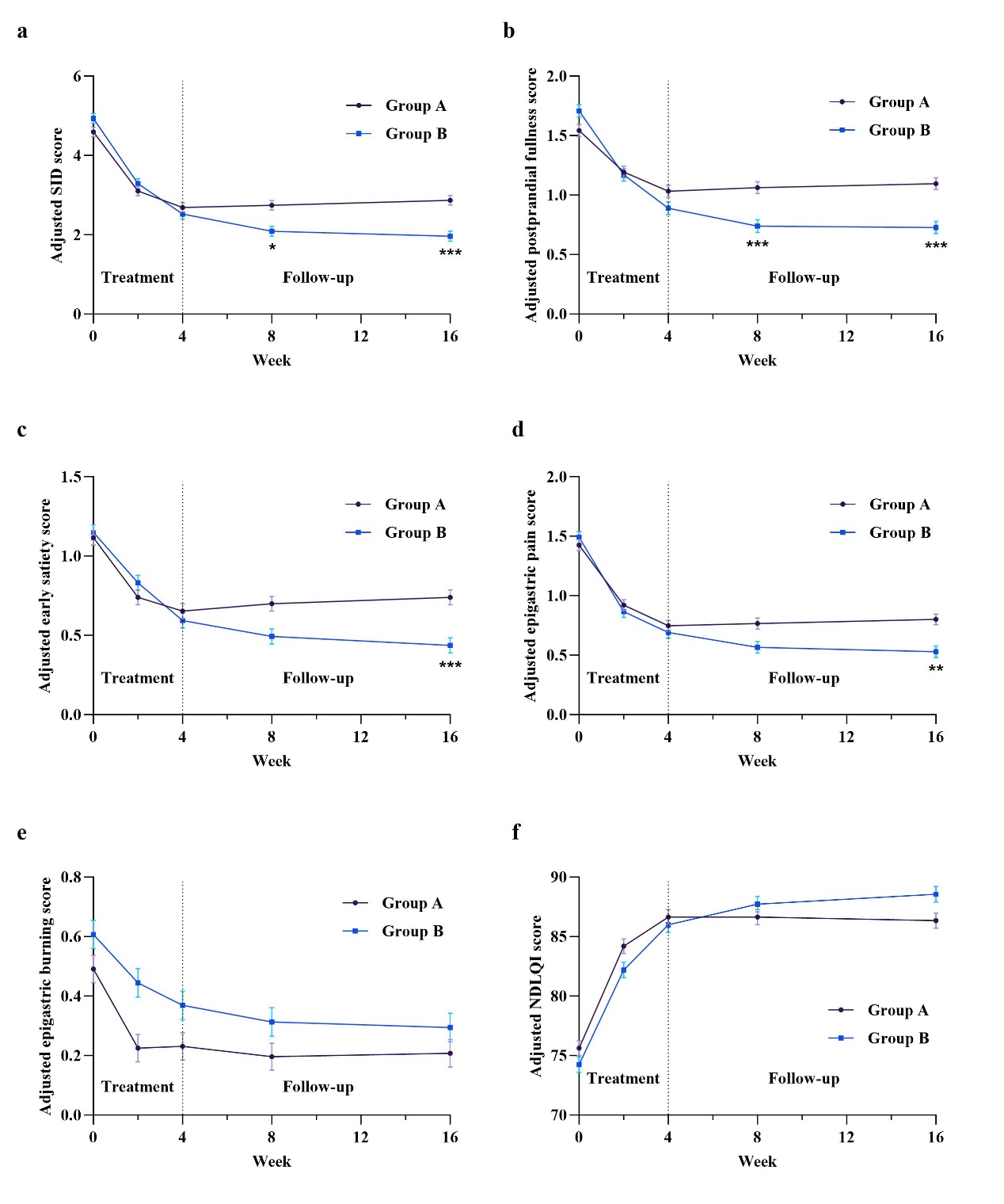


**Figure S4. Adjusted Analysis of Outcomes for EPS-Domain Group Patients**

(a)–(f) show the adjusted scores for SID, postprandial fullness, early satiety, epigastric pain, epigastric burning, and NDLQI, respectively, over the 16‑week period including baseline, 4 weeks of treatment, and 12 weeks of follow‑up. Group A: junior acupuncturist treatment group. Group B: senior acupuncturist treatment group. SID: symptom index of dyspepsia. NDLQI: Nepean dyspepsia life quality index. Comparison between two groups: **^*^***P* < 0.05; **^**^***P* < 0.01; **^***^***P* < 0.001.

# Detailed Acupuncture Prescriptions and Procedures

## Acupoint prescriptions

Prescription A (specific acupoints on the stomach meridian): ST42, ST40, ST36, ST34

Prescription B (non‑specific acupoints on the stomach meridian): ST38, ST35, ST33, ST32

Prescription C (specific acupoints commonly used for FD): BL21, CV12

Prescription D (acupoints on the gallbladder meridian): GB40, GB37, GB36, GB34

Acupuncture procedures (common to all prescriptions, unless otherwise specified)

**Needle insertion and general settings**

Except for CV12, the acupoints were punctured unilaterally by sterile disposable stainless steel needles (40 mm or 25 mm in length and 0.25 mm in diameter; Hwatuo, Suzhou, China, PRC). The needles were alternately placed on the left and right to relieve pain and reduce tolerance. The depth of puncture was described in the protocol, according to the location of the acupoints.

**Manual manipulation and auxiliary needles**

Twirling and rotating, lifting and thrusting manipulation was applied to promote qi arrival. After the arrival of qi, auxiliary needles (13 mm in length and 0.18 mm in diameter) were punctured 2 mm lateral (proximal limbs) to each acupoint at a depth of 2 mm without manual stimulation.

**Electrical stimulation parameters**

Then, a Hans transcutaneous electric acupoint stimulation (TEAS) machine (LH 200A; Nanjing, China) was used. Electrodes were applied on each acupuncture needle and the auxiliary needle for 30 min with a stimulation frequency of 2/100 Hz and an intensity between 0.5 mA and 1.5 mA.

**Treatment schedule**

The acupuncture treatment consisted of 20 sessions over a period of 4 weeks (one session per day, five continual sessions per week, 2 days interval between 2 weeks).

Note: CV12 (in Prescription C) was punctured bilaterally, differing from the unilateral puncture applied to all other acupoints.
